# Neonatal Murine Model of Coxsackievirus A2 Infection for the Evaluation of Antiviral Therapeutics and Vaccination

**DOI:** 10.3389/fmicb.2021.658093

**Published:** 2021-05-28

**Authors:** Wangquan Ji, Luwei Qin, Ling Tao, Peiyu Zhu, Ruonan Liang, Guangyuan Zhou, Shuaiyin Chen, Weiguo Zhang, Haiyan Yang, Guangcai Duan, Yuefei Jin

**Affiliations:** ^1^Department of Epidemiology, College of Public Health, Zhengzhou University, Zhengzhou, China; ^2^Henan Province Center for Disease Control and Prevention, Zhengzhou, China; ^3^School of Public Health, Xinxiang Medical University, Xinxiang, China; ^4^Department of Immunology, Duke University Medical Center, Durham, NC, United States

**Keywords:** hand, foot, mouth disease, coxsackievirus A2, murine model, antiviral, vaccine

## Abstract

Coxsackievirus (CV) A2 has emerged as an important etiological agent in the pathogen spectrum of hand, foot, and mouth disease (HFMD). The symptoms of CVA2 infections are generally mild, but worsen rapidly in some people, posing a serious threat to children’s health. However, compared with enterovirus 71 detected frequently in fatal cases, limited attention has been paid to CVA2 infections because of its benign clinical course. In the present study, we identified three CVA2 strains from HFMD infections and used the cell-adapted CVA2 strain HN202009 to inoculate 5-day-old BALB/c mice intramuscularly. These mice developed remarkably neurological symptoms such as ataxia, hind-limb paralysis, and death. Histopathological determination showed neuronophagia, pulmonary hemorrhage, myofiberlysis and viral myocarditis. Viral replication was detected in multiple organs and tissues, and CVA2 exhibited strong tropism to muscle tissue. The severity of illness was associated with abnormally high levels of inflammatory cytokines, including interleukin (IL)-6, IL-10, tumor necrosis factor α, and monocyte chemotactic protein 1, although the blockade of these proinflammatory cytokines had no obvious protection. We also tested whether an experimental formaldehyde-inactivated CVA2 vaccine could induce protective immune response in adult mice. The CVA2 antisera from the vaccinated mice were effective against CVA2 infection. Moreover, the inactivated CVA2 vaccine could successfully generate immune protection in neonatal mice. Our results indicated that the neonatal mouse model could be a useful tool to study CVA2 infection and to develop CVA2 vaccines.

## Introduction

Hand, foot, and mouth disease (HFMD) is a common infectious disease caused by human *Enteroviruses* (HEVs) belonging to the family *Picornaviridae*. The outbreak of HFMD in the Asia-Pacific region continuously poses a huge threat to infants and young children. HEVs are positive-stranded RNA virus consisting of four species of HEV-A, HEV-B, HEV-C, and HEV-D (Lefkowitz et al., 2018). HEV-A consists of coxsackieviruses (CV) A2-A8, A10, A12, A14, and A16 and enterovirus 71 (EV71) (Oberste et al., 2004). Among these HEVs, EV71, and CVA16 are the two main causative agents responsible for the severe and fatal HFMD (Chen et al., 2007; Mao et al., 2014; Xing et al., 2014). CVA2 caused recent sporadic occurrences and outbreaks of HFMD that have been frequently reported in Asia-Pacific regions, such as China (Hu et al., 2011), Hong Kong (Yip et al., 2013), Taiwan (Yen et al., 2017), Thailand (Chansaenroj et al., 2017), Japan (Ohara et al., 2016), and Korea (Baek et al., 2011). The continuous transmission and evolution of CVA2 have resulted in the genetic polymorphism, and the disease burden caused by CVA2 infections might be highly underestimated (Yang et al., 2018). CVA2 infections are general mild and self-limiting. However, the symptoms of infections in some patients rapidly worsen and become severe with neurological manifestations of encephalomyelitis, acute flaccid paralysis, and cardiorespiratory dysfunction (Bendig et al., 2001; Molet et al., 2016; Yen et al., 2017). CVA2 is a pathogen that is critically involved in herpangina (Chen et al., 2010). In comparison with EV71 frequently detected in fatal cases, the current body of knowledge on CVA2 is extremely limited. Due to the lack of effective antiviral agents, CVA2 infections with severe complications are mainly treated with symptomatic and supportive therapy. With the increase in the morbidity and mortality of CVA2 infection, the pathogenesis of this virus should be urgently studied, and therapeutic agents and vaccines need to be developed.

Animal models serve as an excellent tool for translational research and play a significant role in studying disease pathogenesis and development of vaccines or antiviral drugs. Nowadays, animal models of EV71, CVA16, CVA6, and CVA10 have been well established for the development of vaccines (Ong et al., 2010; Mao et al., 2012; Zhang et al., 2017a, b). However, a suitable animal model for CVA2 infection is still lacking. In the present study, we developed an animal model of CVA2 infection using 5-day-old BALB/c mice. Upon infection, these neonates exhibited neurological presentations, myocarditis, and pulmonary lesions similar to those observed in human patients. By employing this model, we investigated the relevance of increased inflammatory cytokines to the disease severity and the therapeutic effects of cytokine monoclonal antibodies (mAb). Furthermore, we evaluated the immune-protective effect of vaccination in mice with the candidate formaldehyde-inactivated CVA2 vaccine.

## Materials and Methods

### Ethics Statements

The experimental animals were inbred, specific-pathogen-free BALB/c mice (certificate no. DW2019110074). All animal experiments were carried out strictly in accordance with the protocols approved by the Life Science Ethics Review Committee of Zhengzhou University (permission no: ZZUIRB2020-29).

### Cells and Viruses

Human rhabdomoma (RD) cells and African green monkey kidney (Vero) cells (ATCC CCL-81) were cultured in DMEM (Gibco Company, New York, United States) supplemented with 10% fetal bovine serum (FBS, Gibco Company, New York, United States) and incubated at 37°C with 5% CO_2_. A total of 19 stool specimens were collected from children clinically diagnosed with HFMD in the First Affiliated Hospital of Xinxiang Medical College from January 2017 to December 2017. HFMD cases were clinically diagnosed according to the Ministry of Health Diagnostic Criteria^1^. Nineteen children who displayed vesicular rashes on the hands, feet, oral mucosa, or buttock in epidemic seasons were clinically diagnosed with HFMD. In this study, patients with severe complications, including neurological complications (e.g., encephalitis, meningitis, and brain stem encephalitis) and/or cardiorespiratory failure, were considered as having severe HFMD. Stool specimens were homogenized (20% weight/volume) in saline, centrifuged at 4,000 × *g* for 10 min at 4°C, and inoculated 100 μL of each clarified supernatant into RD cells. When cytopathic effect arose, RD cells were harvested for total nucleic acid extraction with a Qiagen Viral RNA extraction kit. The amplification and detection of nucleic acid were carried out using the RT-PCR instrument (SensoQuest). According to the Ministry of Health Diagnostic Criteria^2^, the primers used for viral RNA detection are shown in Supplementary Table 1. Viral genomes were sequenced by standard methods, and the CVA2 strain (We named it HN202009, accession number: MT992622) was used for subsequent animal study. The titers were determined by a median tissue culture infective dose (TCID_50_) assay in accordance with the method of Reed and Muench (Reed, 1938). All CVA2 stocks were subjected to three freeze-thaw cycles, clarified by centrifugation at 4,000 × *g* for 10 min at 4°C, filtered through a 0.22 μm micron filter, and stored at −80°C. The titer was quantified by the Reed–Muench method to be 2.45 × 10^7^ TCID_50_/mL.

### Mice

The BALB/c mice used in this study were obtained from Experimental Animal Center of Zhengzhou University, and all mice were housed in individually ventilated cages (IVC, Tecniplast) in a specific pathogen-free facility of the College of Public Health of Zhengzhou University on a 12 h light/dark cycle with *ad libitum* access to food and water.

### Mouse Infection Experiments

To evaluate the pathogenicity of the CVA2 strain (HN202009) in a neonatal mouse model under different experimental conditions, we developed an animal model of infection based on dosage, inoculation route, and age. For the dose-dependent experiment, 5-day-old BALB/c mice were intramuscularly (i.m.) inoculated with 10-fold serially diluted CVA2 (10^2^–10^7^ TCID_50_ per mouse). To select a suitable inoculation route, we infected 5-day-old BALB/c mice via i.m., intraperitoneally (i.p.), and intracerebrally (i.c.) routes with 10^4^ TCID_50_ of CVA2. To compare the susceptibility of mice with different ages to CVA2, we administered a dose (10^4^ TCID_50_ per mouse) of CVA2 into the mice at different ages (3, 5, and 7 days) via i.m. route. The control mice were inoculated with an equal volume of culture supernatant of RD cells and kept in a separate cage from the infected mice. Each group included 10∼15 animals. The body weight, clinical signs, and survival rates of control or infected mice were recorded for 15 dpi (days post-infection). The grade of clinical disease was scored as follows: 0, healthy; 1, lethargy and inactivity; 2, ataxic; 3, lose weight; 4, hind limb paralysis; 5, dying or death. The control mice were healthy throughout the experiments. Median lethal dose (LD_50_) was calculated using the Reed and Muench method (Reed, 1938).

### Histopathological and Immunohistochemical Analysis

The 5-day-old neonatal mice were i.m. inoculated with 10^4^ TCID_50_ CVA2 strain HN202009. At 7 dpi, control and infected mice were euthanized. The brain, lung, skeletal muscle, spinal cord, and heart samples were obtained and fixed in 10% paraformaldehyde for 48 h. After fixation, paraffin-embedded organs and tissues were cut into 5 μm sections and stained with hematoxylin and eosin (H&E). The expression of CVA2 VP1 in the brain, lung, skeletal muscle, spinal cord, and heart samples of control and infected mice was detected by immunohistochemical (IHC) staining in accordance with a standard immunoperoxidase procedure as described previously (Zhang et al., 2017a). The Mouse CVA2 VP1 mAb used in this study was prepared in our own laboratory.

### Quantitative Real-Time (RT) PCR

Total RNA was extracted from the organs and tissues of control and infected mice using TRIzol reagent (Invitrogen), and cDNA was generated by reverse transcription by using a kit (Yeasen) according to the manufacturer’s instructions. The expression of apoptotic genes of Vero cells such as caspase-8 and -9, and CVA2 VP1 of organs was measured by quantitative RT-PCR (Yeasen) using the instrument (Kubo Tech Co., Ltd.). The primers used for above experiments are listed in Supplementary Table 1. All results were normalized to β-actin expression levels (Liou et al., 2016). Relative expression of tested gene expression was calculated and normalized by the 2^–ΔΔCt^ method.

### Analysis of Tissue Lysates

The brain, lung, and skeletal muscle samples obtained from mice were weighed and quickly lyzed in a cold isolation buffer (Beyotime). The concentration of total protein was determined using a BCA protein assay kit (Biomed) according to the manufacturer’s instructions. The expression levels of IL-6, IL-10, IL-1β, TNF-α, and MCP-1 were measured using enzyme-linked immunosorbent assay (ELISA) kits (Biolegend), and the results were normalized by with the concentration of total proteins in each organ or tissue.

### Western Blot Analysis

Total proteins from Vero cells were extracted using a protein extraction kit (CWbio Company Ltd.) according to the manufacturer’s instruction. For Western blot analysis, samples were resolved on SDS-PAGE and transferred into PVDF membranes. After incubation with primary antibodies, PVDF membranes were washed thrice and incubated with anti-goat secondary antibodies. Membranes were finally washed thrice and developed with an ECL enhanced Chemiluminescence Kit (Absin Bioscience, Inc.). The mouse CVA2 VP1 mAb used in this study was developed in our own laboratory.

### Preparation of Formaldehyde-Inactivated CVA2 Whole-Virus Vaccine and Determination of Anti-CVA2 Antibody Titers

An inactivated CVA2 suspension with a formaldehyde concentration of 1:4,000 (vol/vol) was prepared by mixing 37% formaldehyde (GB/T685-1993) with CVA2 strain HN202009 (2.45 × 10^7^ TCID_50_/mL). The viral suspension was then incubated at 37°C for 3 days (Martin et al., 2003). Viruses were confirmed to be totally inactivated after repeated RD cell culture and blind passage for up to 3 weeks. Five milliliters of the suspension were mixed with Imject^TM^ alum Adjuvant (Thermo) in equal volumes to produce an emulsion. Six-week-old adult female BALB/c mice were inoculated with 200 μL of inactivated CVA2 emulsion via the i.p. route. Blood samples of adult mice were collected 10 days after two immunizations with an interval of 2 weeks. The CVA2 antiserum was separated by centrifugation at 4,000 × *g* for 10 min at 4°C and stored at −80°C until analysis. The geometric mean titer (GMT) of the neutralizing antibody in the CVA2 antiserum was determined by *in vitro* micro-neutralization assays as described previously with slight modifications (Cai et al., 2013). Briefly, the CVA2 antiserum samples were serially diluted (1 to 1:10,000) by 10-folds using MEM containing 2% FBS. Exactly 50 μL of diluted antiserum was mixed with 10^2^ TCID_50_ of CVA2 in 96-well plates and incubated at 37°C for 1 h. Then, 6 × 10^3^ RD cells were added to each well of the 96-well plate and cultured at 37°C under 5% CO_2_. Three days later, the cells were inspected to determine the cytopathic effect (CPE). Neutralization titers were determined as the highest antiserum dilution that could fully protect cells from CPE.

### Evaluation of the Therapeutic Effects of Monoclonal Antibodies Against Different Cytokines

To evaluate the therapeutic effects of mAbs against cytokines, we inoculated a lethal dose of CVA2 into 5-day-old mice (i.m.). After 12 h, these mice were injected i.p. with Ultra-LEAF^TM^ purified neutralizing monoclonal anti-mouse IL-6, TNF-α, and MCP-1 IgG mAb (Biolegend) at 1.33 mg/kg (Khong et al., 2011), 50 mg/kg TNF-α (Scanga et al., 1999; Via et al., 2001), and 10 mg/kg MCP-1 (Morrison et al., 2003) per mouse. Suckling mice from the control group were injected with equivalent purified rat IgG1 (isotype control, Biolegend). All suckling mice were monitored daily upon infection for the occurrence of clinical symptoms and mortality up to day 15 post-infection as the experimental endpoint.

### Effects of Passive Immunization and Maternal Antibody on 5-Day-Old Neonatal Mice

To study the protective effects of passive immunization, we administered CVA2 antiserum with 10-fold serial dilution (1–1:10,000) via i.p. injection into 5-day-old neonatal mice, while control mice were administrated with equal volumes of negative serum (NS) from female mice inoculated with control emulsion (without CVA2). Next day, a lethal dose of CVA2 was inoculated into the mice in both groups by the i.m. route. The clinical signs and survival rates were monitored and recorded daily until 15 dpi. To evaluate the immune-protective effects of maternal antibodies, intraperitoneally inoculated the 6-week-old mice with 200 μL of Imject^TM^ alum adjuvant-inactivated CVA2 emulsion, and the immune response was then strengthened by inoculation with another Imject^TM^ alum adjuvant-inactivated CVA2 emulsion 2 weeks later. After the first immunization, the female and male mice were mated immediately, and the dams were delivered 7∼10 days after the second immunization. Control 6-week-old maternal mice were intraperitoneally inoculated with equal volumes of emulsion (Imject^TM^ alum adjuvant- medium). A lethal dose of CVA2 was i.m. injected into the 5-day-old pups from immunized or control pregnant mice. The clinical signs and survival rates were monitored and recorded until 15 dpi.

### Effects of Active Immunization on Neonatal Mice

Two groups (*n* = 7 per group) of newborn mice were inoculated with 50 μL of inactivated CVA2 whole-virus vaccine via i.p at day 1 and 3, separately. Five-day-old immunized mice (one of the two group) were exposed to a lethal dose of CVA2 (10^4^ TCID50). The mice of another group were inoculated with medium. The unimmunized 5-day-old mice (*n* = 7) were inoculated with an equivalent lethal dose of CVA2. The clinical signs and survival rates were monitored and recorded until 15 dpi.

### Statistical Analysis

Statistical analysis was performed with GraphPad Prism version 8.3 (GraphPad 8.3 Software, San Diego, CA, United States). The Mantel-Cox log rank test was used to compare the survival of different group mice. The results were expressed as the mean ± standard deviation (SD). Differences in the weight, mean clinical score, expression, or transcription levels of cytokines and viral loads were determined using *unpaired student t-test* or *one-way analysis of variance (ANOVA)*. A *P*-value of < 0.05 after two-tailed *t* testing was regarded as significant.

## Results

### Identification of CVA2 Strains in Clinical Isolates From HFMD Patients

**FIGURE 1:**
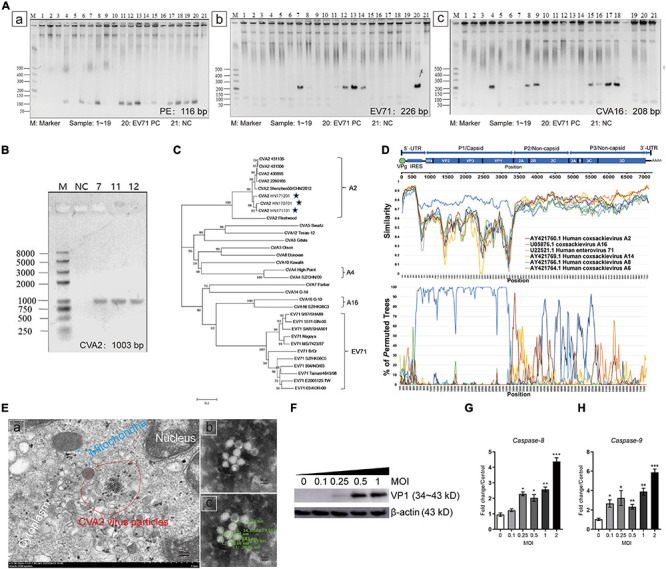
Identification of CVA2 strains in clinical isolates from HFMD patients. **(A)** PCR analysis of 19 patient samples. a. samples 4, 6, 7, 8, 9, 11, 12, 13, 14, 15, 16, 17, 18, and 19 showed positive PE nucleic acid; b. Samples 7, 8, 11, 12, 13, and 14 were positive for EV71 nucleic acid; and c. Samples 4, 6, 8, 9, 15, 16, 17, and 18 were positive for CVA16 nucleic acid. **(B)** The PCR products (1,003 bp) of samples 7, 11, and 12. **(C)** A genetic evolutionary tree of samples 7, 11, and 12 VP1 (1,003 bp) by using the neighbor-joining method. **(D)** Recombination analysis of the full-length genome of HN202009 by using the Simplot. **(E)** Transmission electron micrograph of the infected Vero cells and inactivated CVA2 virions. a. Virus inclusion bodies in infected Vero cells; b and c. Spherical particles of the CVA2 virions with a diameter of 26∼35 nm. **(F)** The relative expression of VP1; and **(G,H)**. The expression of caspase-8 and caspase-9 RNA in the infected cells with increased doses of CVA2. Positive control, PC; negative control, NC. *P < 0.05; **P < 0.01; ***P < 0.001.

A total of 19 patients diagnosed with HFMD were included in this study, and the clinical features are shown in Supplementary Table 2. Among them, 11 (57.89%) patients were severely diagnosed by clinicians. Eight (42.11%), three (15.79%), and five (26.32%) patients of these 19 patients were positive to enterovirus (EV) infection, EV71 IgM, and C-reactive protein (CRP), respectively, based on relevant clinical tests. Stool specimens from these patients were collected for further laboratory testing. The clarified supernatants of stool specimens were used to inoculate the RD cell line for four generations in blind passage. As shown in Supplementary Figure 1, after 48 hpi, all isolates from 19 specimens could induce CPE in RD cells. We used the primers of pan enteroviruses (PE), EV71, and CVA16 for nucleic acid test according to the Ministry of Health Diagnostic Criteria (Supplementary Table 1). Our results showed that strains #4, #6-#9 and #11∼#19 were PE positive (Figure 1Aa), strains #7, #8 and #11∼#14 were EV71 positive (Figure 1Ab), and strains #4, #6, #8, #9 and #15∼#18 were CVA16 positive (Figure 1Ac). Next, the RT-PCR products from above were sequenced. Notably, sequence alignment showed that the clinical isolates, which were EV71 positive (#7, #11, and #12), were actually CVA2 strains (Supplementary Table 3). We designed a new pair of primers based on the sequence of CVA2 VP1 genome (Supplementary Table 1) to confirm these findings. As shown in Figure 1B and Supplementary Table 3, these three strains were indeed CVA2 strains. Phylogenetic tree analysis based on the capsid protein P1 of CVA2, CVA4, CVA16, and EV71 strains identified worldwide also showed A2 type of these CV strains (Figure 1C). The nucleotide sequences of the VP1 genome in these three CVA2 strains were deposited in the GenBank database under accession number, MT847604, MT847605, and MT847606, respectively (We named them HN171201, HN170701, and HN171101, respectively.). In this study, we selected strain #11 (HN171101, accession number: MT847606) for subsequent animal experiments based on the results from the induced CPE in RD cells. The full-length genomic sequence of #11 strain was deposited in the GenBank database under accession number MT992622. The phylogenetic tree analysis of HN202009 together with the strains that have the highest homologous sequence in the NCBI database by using the neighbor-joining method in the MEGA-X software (Supplementary Figure 2). Subsequently, we performed recombination analysis of HN202009 strain (Figure 1D) and found that HN202009 exhibited the highest degree of similarity to CVA2 Fleetwood (AY421760.1) in the capsid region with approximately 80% nucleotide identity. However, the boot scanning graph showed a sound phylogenetic relationship in the non-capsid region between HN202009 and the other EVs, indicating possible recombination events. HN202009 was most closely related to AY421760.1 in the capsid genome, which was consistent with the Simplot analysis results. We detected CVA2 virus particles in infected Vero cells (Figure 1Ea), and the shape of inactivated CVA2 viruses was round or oval with a diameter of 26∼35 nm (Figures 1Eb,c). As shown in Figure 1F, the expression of VP1 was increased after CVA2 infection at the indicated doses and exhibited a marked dose-dependent manner. We also found that CVA2 infection could induce cell apoptosis with the increased levels of caspase-8 and caspase-9 mRNA (Figures 1G,H). Our results suggest the outbreak of CVA2 in Henan Province, China and a natural recombinant of CVA2 (HN202009) infection has a correlation with HFMD severity.

### Establishment of a Mouse Model of CVA2 Infection

**FIGURE 2:**
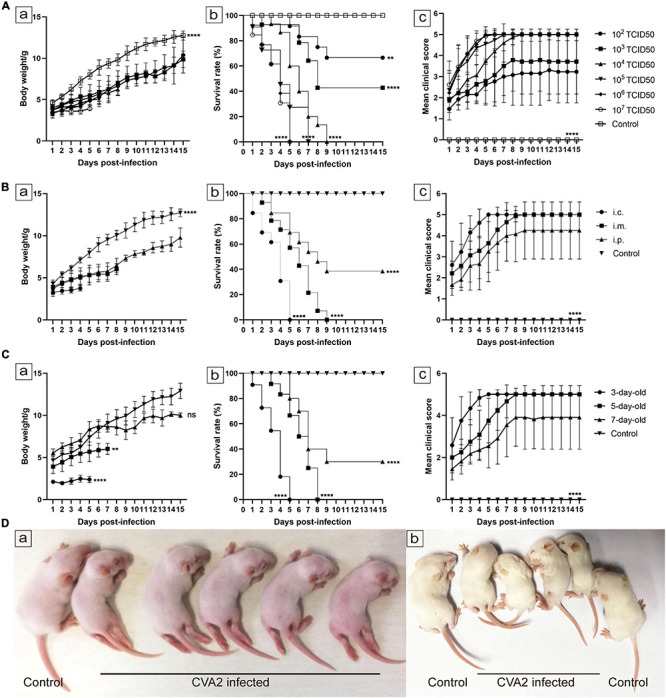
Establishment of the CVA2 infection mouse model. **(A–C)** Body weights, survival rates, and mean clinical scores of mice. Five-day-old BALB/c mice (*n* = 10∼15 per group) were i.m. inoculated with different doses of CVA2 (10^2^∼10^7^ TCID_50_/mouse, respectively). Control animals were administered culture medium instead of virus. The body weights **(A-a,B-a,C-a)**, survival rates **(A-b,B-b,C-b)**, and clinical scores **(A-c,B-c,C-c)** in each group of neonatal mice were measured. **(D)** Two representative pictures of clinical signs (weight loss, reduced mobility, ataxia, and single or double hind limb paralysis) induced by CVA2 in mice. a. 5 dpi and b. 7 dpi. **P < 0.01; ****P < 0.0001; ns, non-significant result.

To establish a mouse model for CVA2 infection, we inoculated 5-day-old neonatal mice via i.m. with 10-fold serially diluted CVA2 (10^2^–10^7^ TCID_50_ per mouse) and observed the development of clinical signs. Mice inoculated with CVA2 at doses of 10^7^ and 10^6^ TCID_50_ rapidly died within 5 days. When the infectious dose was reduced to 10^3^ or 10^2^ TCID_50_ per mouse, these mice showed neurological symptoms, such as ataxia and paralysis, or death at 4–5 dpi, and the ultimate survival rates were 42.86 and 61.54%, respectively. In comparison with the other infectious doses, the mice infected with the challenge dose of 10^4^ TCID_50_ exhibited typically neurological symptoms at 2 dpi and all died at 9 dpi (Figure 2A). These results showed that CVA2-infected mice exhibited dose-dependent pathogenesis and mortality. The LD_50_ of CVA2 strain HN202009 was 3.63 × 10^2^ TCID_50_. Hence, the titer of 10^4^ TCID_50_/mouse was selected as the optimal inoculation dose (approximately 28 LD_50_). To determine the effect of inoculation route on CVA2 susceptibility, we infected 5-day-old BALB/c mice via i.p., i.c, or i.m. routes with a lethal dose of CVA2 (10^4^ TCID_50_). Both mice inoculated via the i.c. and i.m. routes became sick at 1 dpi and resulted in 100% mortality. However, the i.c route was unsuitable for antiviral studies because of the rapid disease process and short survival time. Compared with i.c. and i.m. routes, mice inoculated with CVA2 via i.p. remained resistant to disease (Figure 2B). The i.m. route was finally selected as the standard route of CVA2 infection in the subsequent experiments. To determine the age susceptibility to CVA2 infection, we administered 10^4^ TCID_50_ CVA2 to mice with different ages (3, 5, and 7 days) via the i.m. route. CVA2 infection resulted in a rapid disease onset and a short survival time in 3-day-old mice. Mice younger than 7 days were more susceptible to CVA2 infection, implying age dependency. The average clinical scores of the 5-day-old mice were more than 4 after 5 dpi, and all infected mice died between 3 and 8 dpi (Figure 2C). Therefore, the 5-day-old BALB/c mice were selected as animal models in the subsequent experiments. Control mice inoculated with culture supernatants of RD cells were healthy throughout the experiment. Clinical signs of weight loss, reduced mobility, and neurological signs, such as ataxia, and limb paralysis in CVA2-infected mice were shown in Figure 2D, and these neurological manifestations were similar to those with human infections.

### Histopathological Determination in CVA2-Infected Mice

**FIGURE 3:**
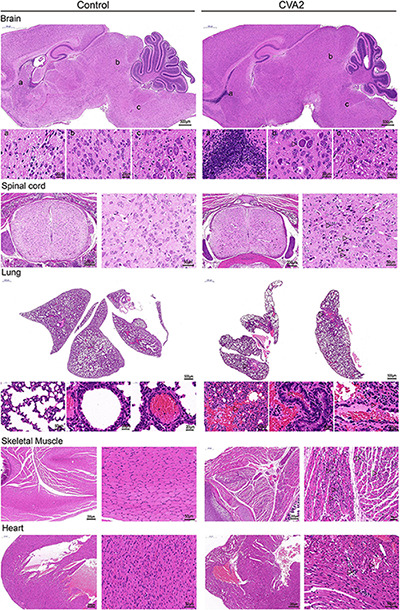
Histopathological changes in CVA2-infected mice. In the brain tissue and spinal cord, neurophagy and lymphocyte infiltration occurred. The lungs presented inflammatory hyperemia characterized with massive erythrocytes leakage. The skeletal muscles exhibited myofiberlysis, necrosis, and inflammatory cell infiltration. Severe viral myocarditis was observed, characterized with lymphocytic and mononuclear cell infiltration and myocardial rupture. In control mice, the upper organs or tissues were almost unaffected. All experiments were repeated for four times.

To identify the pathological features of CVA2-infected mice, we performed H&E staining (Figure 3) of major visceral organs or tissues at 7 dpi. We observed cellular damage (such as degeneration and neuronophagia) and inflammatory changes (such as gliosis and perivascular cuffing) in the brains and spinal cords of infected mice with clinical signs. We also observed severe pathological changes in lungs and skeletal muscles derived from CVA2-infected mice. The lungs presented inflammatory hyperemia, characterized with massive erythrocytes leakage, which was typical clinical manifestation of pulmonary hemorrhage in human infections. The skeletal muscles exhibited myofiberlysis, necrosis, and inflammatory cell infiltration. In addition, we observed viral myocarditis characterized with lymphocytic and mononuclear cell infiltration and myocardial rupture. In control mice, the relevant organs or tissues were almost unaffected.

### Viral Replication in CVA2-Infected Mice

**FIGURE 4:**
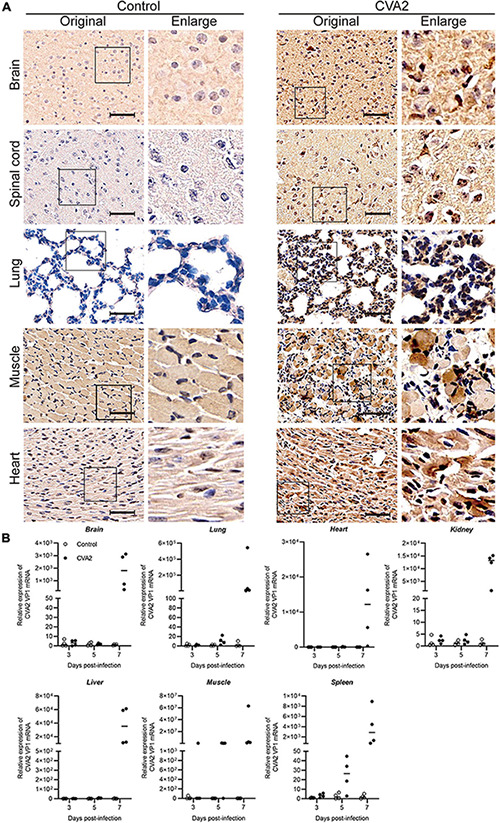
Viral distribution in the tissues and organs of CVA2-infected mice. Immunohistochemical staining and CVA2 viral loads of infected 5-day-old mice after intramuscular challenge with lethal doses of CVA2 or medium (mock control). **(A)** The viral antigen was diffusely distributed in all the isolated tissues; no antigen was detected in the control group. Magnification: 500×. All experiments were repeated for four times. **(B)** The viral loads of different organs (brain, lungs, heart, liver, spleens, kidney, and limb muscles) from mice infected with CVA2. Results are expressed as numbers of viral RNA copies normalized by β-actin from the same organ/tissue (n = 4 per group).

IHC staining of major organs or tissues (Figure 4A) was performed at 7 dpi to determine the antigen distribution in CVA2-infected mice. Viral antigen (CVA2 VP1) was detected in the affected neurons in the brain and spinal cord samples, and alveolar cells in lungs, and muscle fiber cells in skeletal muscles, and cardiomyocytes in heart, derived from CVA2-infected mice. The control mice did not have any VP1 antigens in above organs or tissues as expected. Next, viral loads, which were expressed as CVA2 RNA level in multiple organs or tissues (Figure 4B), were determined at 3, 5, and 7 dpi by using quantitative RT-PCR. Viral loads in the brain, lung, heart, kidney, liver, skeletal muscle, and spleen samples from CVA2-infected mice reached the maximum at 7 dpi. Consistent with the severe pathological changes in the skeletal muscles, at the early stages of 3 dpi, viral replication with extremely high viral load was only detected in skeletal muscles. In addition, the lung, heart, kidney, liver, and spleen samples from CVA2-infected mice had comparably high viral loads at later time points. Collectively, viral replication was detected in multiple organs or tissues from CVA2-infected mice, and skeletal muscle might be the main replication site, thus supporting the pathological changes in corresponding tissues or organs.

### Expression of Inflammatory Cytokines in the Tissue Lysates of CVA2-Infected Mice

**FIGURE 5:**
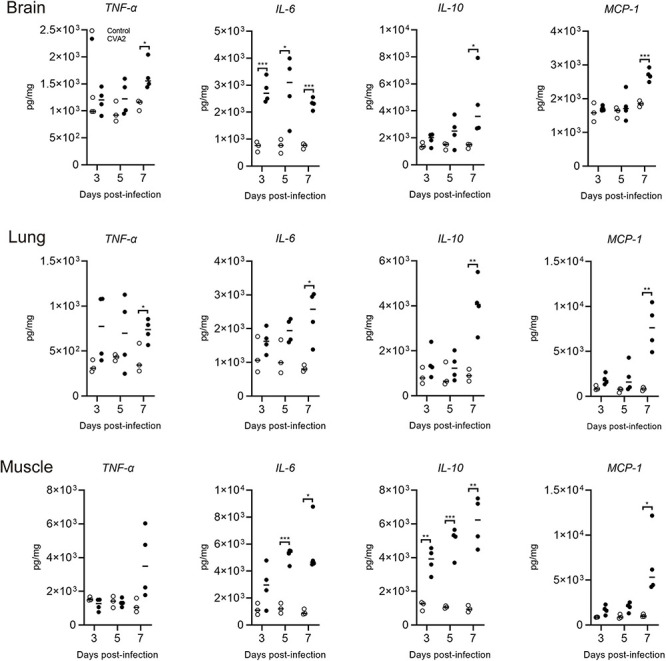
Expression of tissue grinding lysate proinflammatory cytokines in CVA2-infected mice. The expression levels of IL-6, IL-10, TNF-α, and MCP-1 in tissue grinding lysate (brain, lung, and muscle) of the 5-day-old neonatal mice via i.m. inoculated with lethal doses of HN202009 at 3, 5, and 7 dpi were determined using mouse ELISA detection kits (*n* = 4). *P < 0.05; **P < 0.01; ***P < 0.001.

To examine the involvement of inflammatory reaction in the process of CVA2 infection, we analyzed the expression of inflammatory cytokines in tissue lysates at 3, 5, and 7 dpi by ELISA. As shown in Figure 5, the transcription levels of TNF-α, IL-6, IL-10, and MCP-1 in the tissue lysates of brains, lungs, and skeletal muscles derived from CVA2-infected mice were all significantly induced at 7 dpi (*P* < 0.05). They were induced at the early stage of infection and were increased rapidly. The protein expression level of IL-1β in the tissue lysates was below detection. It is possible that these pro-inflammatory cytokines, such as TNF-α, IL-6, and MCP-1, might play important roles in the pathogenesis of CVA2 infection.

### Therapeutic Effect of Anti-cytokine mAbs for CVA2 Infection *in vivo*

**FIGURE 6:**
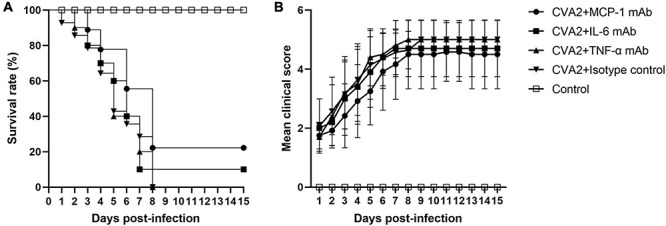
The therapeutic effect of mAb treatment in the CVA2 infection mouse model. Five groups (n = 7 per group) of 5-day-old mice were inoculated i.m. with 10^4^ TCID_50_/mouse of CVA2. After 12 h, mice were injected i.p. with Ultra-LEAF^TM^ purified neutralizing monoclonal anti-mouse IL-6 (1.33 mg/kg), TNF-α (50 mg/kg), and MCP-1 (10 mg/kg) IgG mAb. Suckling mice from the control group were injected with equivalent purified rat IgG1 (isotype control). The treated mice were observed daily for survival rates **(A)** and clinical scores **(B)** and there was no significant statistical difference compared with group CVA2 + Isotype control.

To access the therapeutic roles of anti-cytokine mAb *in vivo*, we inoculated 5-day-old neonatal mice via i.m. with 10^4^ TCID_50_ CVA2 and administered with isotype control, anti-TNF-α, anti-IL-6, and anti-MCP-1 mAbs via the i.p. route at 12 h post infection. As shown in Figures 6A,B, anti-IL-6 mAb and anti-MCP-1 mAb treatment seemed to improve the survival rate and delayed disease onset, but the differences had no statistical significance (*P* > 0.05). Additionally, anti-TNF-α mAb treatment showed no effect on the survival rate and the mean clinical scores of CVA2-infected mice. Therefore, the blockage of pro-inflammatory cytokines failed to stop disease progression induced by CVA2 infection.

### Effects of Passive Immunization and Maternal Antibody on 5-Day-Old Neonatal Mice

**FIGURE 7:**
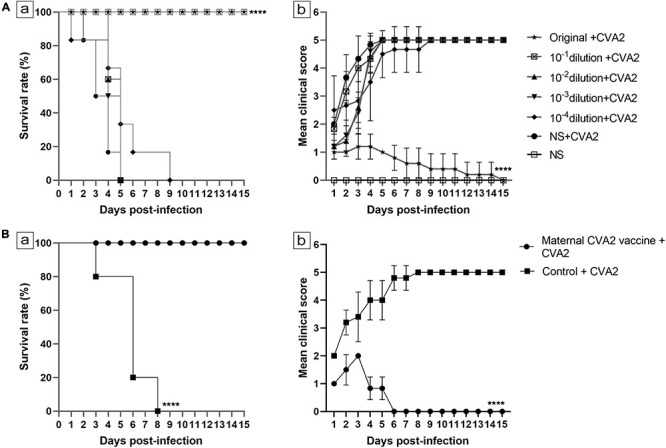
Effects of passive immunization and maternal antibody on 5-day-old neonatal mice. **(A)** Effects of passive immunization. **(B)** Effects of maternal antibody. The survival rates **(A-a,B-a)** and clinical scores **(A-b,B-b)** were monitored and recorded daily until 15 dpi (n = 10−15 per group). The Mantel-Cox log rank test was used to compare the survival of the pups in each antiserum group and that of the pups in the control group at 15 dpi. ****P < 0.0001.

Next, we would like to study whether therapeutic antibodies could be used to control CVA2 infection. First, CVA2 antiserum was collected at 10 dpi after two immunizations of mice with inactivated CVA2 vaccine and used in a microneutralization assays. The GMT of CVA2 antibody (GMT = 1:1,000) was calculated by applying 10-fold serial dilutions of the CVA2 antiserum to RD cells. To evaluate the therapeutic effect of the CVA2 antiserum on the mice, we passively transferred 50 μL of the CVA2 antiserum at original and dilutions of 1:10, 1:100, 1:1,000, and 1:10,000 or the negative serum (NS) into suckling mice, followed by i.m. inoculation with 10^4^ TCID_50_ CVA2. As shown in Figure 7A, mice received NS and diluted CVA2 antiserum started to present clinical signs of limb paralysis at 1 dpi and all died at 9 dpi. No protective effect was observed in mice with the administration of diluted CVA2 antiserum. By contrast, all mice administered with original CVA2 antiserum survived, and no neurological symptoms were observed, indicating that CVA2 antiserum could provide 100% protection to the infected neonatal mice.

To determine the effect of maternal antibody, we immunized 6-week-old female mice twice with the inactivated CVA2 or the control emulsion. After delivery, the 5-day-old newborn mice were i.m. inoculated with 10^4^ TCID_50_ CVA2. The experimental group remained normal during the observation period with a 100% survival rate and showed no clinical signs of ataxia and limb paralysis, indicating that the maternal antibody could provide immuno-protection for pups. However, the clinical symptoms of the control group were severe, and all the mice died at 3∼8 dpi (Figure 7B). Together, passive immunization and maternal antibody provided good immune protection in newborn mice.

### The Active Immunization With Inactivated CVA2 Whole-Virus Vaccine Protects Neonatal Mice Against Infection

**FIGURE 8:**
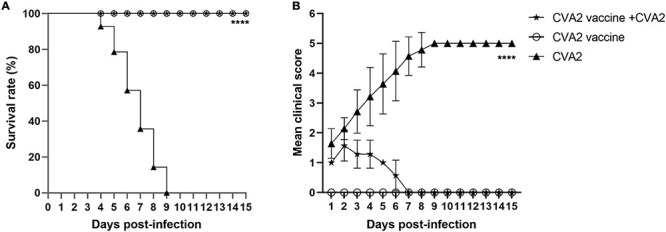
The active immunization with inactivated vaccine protects pups against viral challenge. The inoculated mice were monitored daily for survival rates **(A)** and clinical scores **(B)** for 15 days. The log-rank (Mantel-Cox) test was used to compare the survival curve between vaccine group and the negative control group. ****P < 0.0001.

To study whether active immunization could protect neonates, we inoculated newborn mice with 50 μL of inactivated CVA2 whole-virus vaccine via i.p at day 1 and 3, respectively and then exposed 5-day-old immunized mice to a lethal dose of CVA2 (10^4^ TCID_50_). As shown in Figure 8A, after CVA2 infection, the survival rate of the mice in the active immunization group was 100%, while the mice without vaccination totally died at 9 dpi. The vaccinated mice only presented mild clinical symptoms with 1 or 2 clinical score within 6 days and then stayed normal (Figure 8B). By contrast, the unvaccinated mice showed clinical signs at 2 dpi, and all developed into severe or death at 4∼9 dpi. These data indicated that the inactivated CVA2 whole-virus vaccine could generate immune protection in neonatal mice.

## Discussion

Due to the wide application of EV71 inactivated vaccine in China, EV71 is no longer the leading cause of HFMD (Huang et al., 2018); however, frequent outbreaks of CVA2-related HFMD have been reported worldwide, and the emergence of fatal cases is closely watched. Considering the limited knowledge of this emerging virus, a sensitive and reproducible animal model of CVA2 infection is needed to study the pathogenesis of this virus and to develop therapeutic vaccines and antiviral drugs. To the best of our knowledge, an animal model of CVA2 infection has not been established. In the present study, we introduced a neonatal mouse model for CVA2 infection by i.m. inoculating 5-day-old BALB/c mice with a lethal dose of CVA2 strain (HN202009). The infected mice exhibited ataxia, limb paralysis, tachypnea, and even death, which were similar to the clinical signs of human infections. Moreover, the results of histopathological examination showed neuronal damage and gliosis in the central nervous system, pulmonary hemorrhage, myofiberlysis, and necrosis in skeletal muscle and leukocyte infiltration in myocardial tissue. The results of IHC staining and quantitative RT-PCR indicated CVA2 replication in multiple organs or tissues, and CVA2 exhibited strong tropism to the skeletal muscles. Furthermore, CVA2 infection induced expression of inflammatory cytokines in target organs or tissues; however, the blockade of pro-inflammatory cytokines had no clear therapeutic effects in mice after CVA2 infection. We also used this model to evaluate the protective effects of maternal antibodies, and passive and active immunization with an inactivated whole-virus vaccine, showing good immune protection in neonatal mice. Thus, our animal model can be employed for further development of antiviral drugs and vaccines, and the pathogenesis of CVA2.

The EV71 and CVA16 vaccines were developed with the help of several animal models (Ong et al., 2010; Dong et al., 2011; Yao et al., 2019; Yang et al., 2020). In the present study, we accidentally found that some EV71-positive patients with HFMD were actually infected with CVA2, suggesting that the specificity of laboratory general primers designed for EV71 test should be improved. CVA2 is an emerging pathogen of HFMD in the world, and we first reported the outbreak of CVA2 in Henan Province, China. Natural recombination is a frequent event in human enterovirus A evolution (Hu et al., 2011). Our data also showed that possible recombination occurred in the non-capsid region with other HEVs. The pairwise comparison of VP1 nucleotide sequences of the same serotypes revealed high intra-type variation of CVA2 isolates (84.6–99.3% nucleotide identity) (Park et al., 2012). Further research is needed to determine why EV71 general primers were able to amplify the VP1 of CVA2.

CVA2 can cause severe respiratory symptoms and different neurological manifestations in humans, such as encephalomyelitis and polio-like flaccid paralysis (Yip et al., 2013; Yen et al., 2017). Some severe infections were suddenly died without obvious warning (Yip et al., 2013; Molet et al., 2016). Clinical reports also indicated fatal myocarditis and pulmonary edema in CVA2 infections (Bendig et al., 2001). In the present study, a clinical CVA2 strain was employed to inoculate 5-day-old BALB/c mice by i.m., and the mice developed significant clinical signs, including weight loss, reduced mobility, ataxia, and limb paralysis, with significant pathology in the brain, spinal cord, heart, skeletal muscle, and lung samples of infected mice in the moribund state. Histological and viral examinations demonstrated viral replication in the brain, spinal cord, skeletal muscle, lung, and heart samples. These results are very similar to those from other animal models of EV71, CVA16, CVA6, and CVA10 (Liu et al., 2014; Wang and Yu, 2014; Zhang et al., 2017a, b), suggesting that the tissue damage induced by CVA2 is related to viral replication. Moreover, unlike the other animal models of HEVs, viral myocarditis and pulmonary congestion were observed in neonatal mice after CVA2 infection. Our findings highlighted the similarities in neurological complications and in myocardial and lung injury observed in humans and mice. Furthermore, the age-dependent susceptibility of neonatal mice to CVA2 infection reflects the human situation, where children younger than 5 years old are more susceptible for HEV infection (Solomon et al., 2010). Nonetheless, some differences remain between mice and humans. Enteroviruses are mainly transmitted via the fecal–oral route (Solomon et al., 2010). In comparison with human infections, mice were less susceptible to oral infection and did not display typical cutaneous lesions found in humans (Ooi et al., 2010; Fujii et al., 2013; Yang et al., 2016). Similar to other enterovirus infection models, skeletal muscle exhibited severe damage and had high viral titers of viruses, which were not observed in humans. Our results also indicated that skeletal muscle was the major site of viral replication during the early stage of CVA2 infection. Therefore, whether limb paralysis was caused primarily by skeletal muscle or CNS damage remains to be determined. Skeletal muscles are known to support persistent enterovirus infection and provide a viral source of entry into the CNS during poliovirus infection. In this study, mice with i.c. inoculation rapidly developed into limb paralysis, while the development of severe illness in mice with i.p. or i.m. inoculation was delayed. Hence, CVA2 could somehow spread to the brain via the spinal cord with the increase of virus propagation in the back muscle of mice, leading to disease manifestations such as flaccid paralysis.

A cytokine storm is an excessive immune response and a complication that significantly contributes to HFMD severity (Solomon et al., 2010). Extremely high levels of several cytokines and chemokines have been reported for patients with severe HFMD patients (Solomon et al., 2010). Likewise, the levels of inflammatory cytokines of IL-6, IL-10, TNF-α, and MCP-1 were significantly elevated in the tissue lysates of brain, lung, and skeletal muscle samples obtained from CVA2-infected mice. To explore the actual roles of the above-mentioned proinflammatory cytokines in the animal model, we administered anti-TNF-α, anti-IL-6, and anti-MCP-1 mAbs into neonatal mice that were inoculated with a lethal dose of CVA2. Although anti-IL-6 and anti-MCP-1 mAbs improved the survival rate and delayed the onset of clinical symptoms, no statistical significance was observed. Some researchers have proposed that sustained high levels of IL-6 alone can cause severe tissue damage (Mihara et al., 2012; Tanaka et al., 2014; Hunter and Jones, 2015). The administration of anti-IL-6 neutralizing antibodies after the onset of clinical symptoms successfully improved the survival rates and reduced the clinical scores of the EV71-infected mice (Khong et al., 2011). Except for IL-6, the two other pro-inflammatory cytokines (TNF-α and MCP-1) also play important roles in the occurrence and development of many diseases (Kalliolias and Ivashkiv, 2016; Udalova et al., 2016; Franca et al., 2017; Bianconi et al., 2018; Georgakis et al., 2019). Therefore, whether cytokine mAbs have any therapeutic effects on CVA2 needs further investigation.

Immunization is the most effective tool for the control of HFMD epidemics. Thus far, no effective treatment regimen has been developed for HFMD, and only i.v. immunoglobulin has been licensed as an anti-EV71 therapy; however, its efficacy has not been demonstrated (Wang et al., 2006). An important part for the development of a CVA2 vaccine is the verification of protective functions of anti-CVA2 serum *in vivo* similar to those of EV71 antiserum (Li et al., 2014; Zhu et al., 2014). In the current study, we i.p. inoculated neonatal mice with CVA2 antiserum to examine the protective efficacy of passive immunization. We found that the CVA2 antiserum could inhibit the CPE induced by CVA2 *in vitro*. Original antiserum showed 100% protection against a lethal viral challenge, indicating that neutralizing antibodies may play an essential role for *in vivo* protection but required a higher quantity to achieve protection. The specific CVA2 neutralizing antibody had a protective efficacy with a significant dose-response effect in neonatal mice. After delivery of immunized adult female mice, the 5-day-old newborn mice were i.m. inoculated with 10^4^ TCID_50_ CVA2. The pups from immunized mothers remained normal during the observation period with a 100% survival rate and showed no clinical signs of ataxia and limb paralysis, indicating that the specific maternal antibody vertically transferred from mothers across the placenta to pups could provide excellent protection against CVA2 challenge. EV infection or immunization can induce generation of neutralizing antibodies, and the positive rate of neutralizing antibodies was high in babies or infants living in epidemic areas (Mao et al., 2010). Our results suggested that the candidate CVA2 vaccine induced sufficiently high levels of protective neutralizing antibodies in adult female mice and protected their pups from infection by vertical transmission (Fu et al., 2016). The EV71 vaccine can provide protection against EV71-associated HFMD or herpangina in infants and young children (Zhu et al., 2013, 2014). Although the immune system of neonatal mice is not fully developed, and a complete, fully functional immune response is hard to induce. Our results clearly indicated that the active immunization with inactivated CVA2 whole-virus vaccine could protect the neonatal mice against a lethal dose of CVA2. Inactivated vaccines could generate immune protection in 1-day-old neonatal mice despite the relatively low antibody titers in neonatal mice (Zhang et al., 2018). The phase III clinical trial indicated that the low antibody titer (1:16) can effectively prevent EVA71-associated HFMD or herpangina (Zhu et al., 2014), which may be associated with the high levels of B lymphocyte humoral immune responses stimulated by the secretion of IL-4 (Kohlmeier and Woodland, 2009; Zhang et al., 2018). The difference in neutralizing epitopes or cross-protective capacity with other EVs requires further investigation.

In summary, we have first developed a sensitive and reproducible model for CVA2 infection. By using this animal model, we determined the histopathology of CVA2 infection and studied the relationship between the abnormal expression of proinflammatory cytokines and disease severity. Importantly, passive immunization with CVA2 antiserum and maternal antibody provided good immune protection. Furthermore, active immunization with the inactivated CVA2 whole-virus vaccine could generate immune protection in neonatal mice. Taken together, this mouse model of CVA2 infection provides a reliable tool for the development of antiviral drugs or vaccines, which will be beneficial for the prevention and control of CVA2-associated HFMD.

## Data Availability Statement

The datasets presented in this study can be found in online repositories. The names of the repository/repositories and accession number(s) can be found in the article/Supplementary Material.

## Ethics Statement

The animal study was reviewed and approved by the Life Science Ethics Review Committee of Zhengzhou University (permission no: ZZUIRB2020-29).

## Author Contributions

WJ, YJ, LQ, and GD conceived and designed the research. WJ, LQ, LT, PZ, RL, and GZ directly participated in the experiment. WJ and YJ drafted the manuscript. LT, SC, WZ, and HY participated in the design of study and modification of English grammar. All authors read and approved the final manuscript.

## Conflict of Interest

The authors declare that the research was conducted in the absence of any commercial or financial relationships that could be construed as a potential conflict of interest.

## Footnotes

1 http://www.nhc.gov.cn/yzygj/s3593g/201306/6d935c0f43cd4a1fb46f8f71acf8e245.shtml

2 http://www.nhc.gov.cn/jkj/s3577/200805/e73df45b7b1549188b1d4e1efd604da9.shtml

## Supplementary Material

Supplementary Figure 1 Clinical isolates induced CPE in RD cells. After 48 hpi, all isolates from 19 specimens could induce CPE in RD cells.

Supplementary Figure 2 Phylogenetic tree analysis of CVA2 strain HN202009 combined with a few of strains with the highest homologous sequence in NCBI by using the neighbor-joining method with the help of MEGA-X. •; CVA2 strain prototype (AY421760.1); ■; Virus strains used to establish animal models (MT992622).
