# Supplementary figures and images for: Neonatal Murine Model of Coxsackievirus A2 Infection for the Evaluation of Antiviral Therapeutics and Vaccination
Source: Front Microbiol. 2021 May 28;12:658093. doi: 10.3389/fmicb.2021.658093 (PMC8192712; doi:10.3389/fmicb.2021.658093)

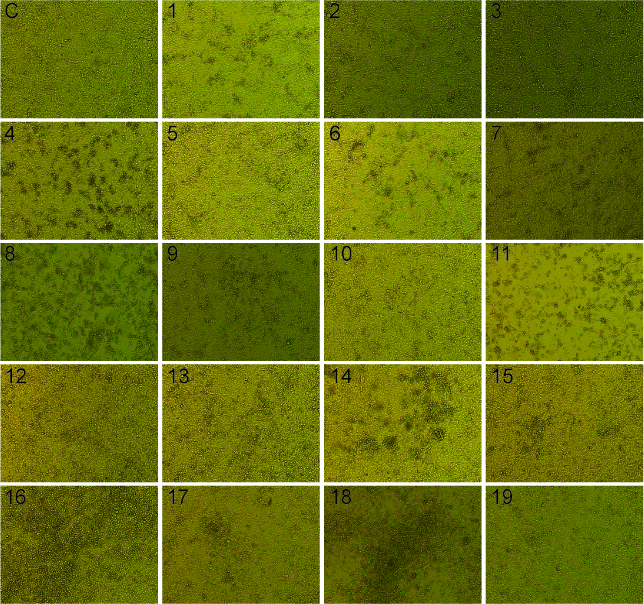

Supplement: Supplementary Figure 1 — Clinical isolates induced CPE in RD cells. After 48 hpi, all isolates from 19 specimens could induce CPE in RD cells. [file Image_1.TIF]

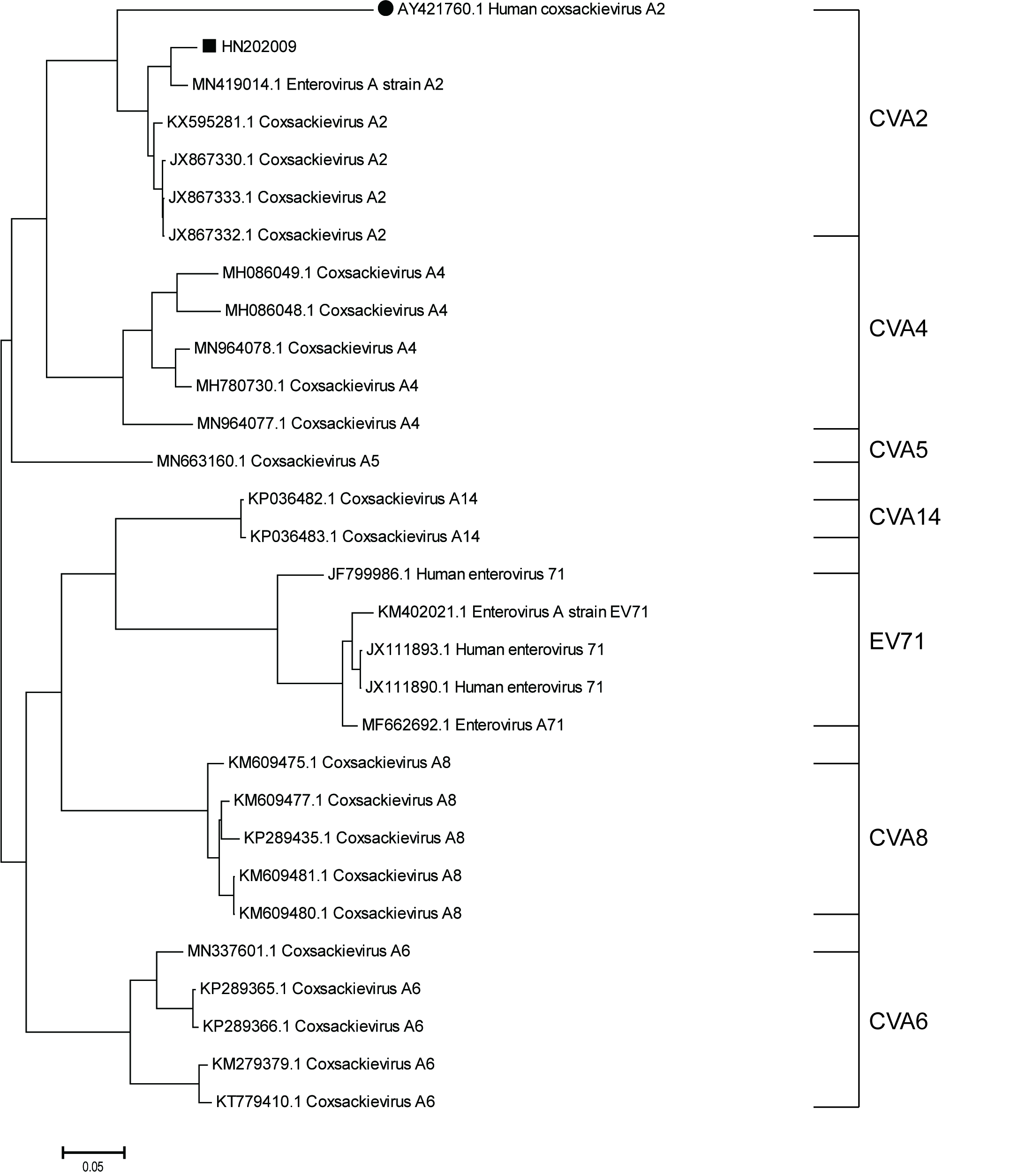

Supplement: Supplementary Figure 2 — Phylogenetic tree analysis of CVA2 strain HN202009 combined with a few of strains with the highest homologous sequence in NCBI by using the neighbor-joining method with the help of MEGA-X. •; CVA2 strain prototype (AY421760.1); ■; Virus strains used to establish animal models (MT992622). [file Image_2.TIF]
