# Supplementary material for: Neonatal Murine Model of Coxsackievirus A2 Infection for the Evaluation of Antiviral Therapeutics and Vaccination
Source: Front Microbiol. 2021 May 28;12:658093. doi: 10.3389/fmicb.2021.658093 (PMC8192712; doi:10.3389/fmicb.2021.658093)
Supplement: Supplementary file 3 [file Table_1.DOCX]

**Table S1 Primers Sequences**

| Gene | Forward | Reverse | Product lengths (bp) |
| --- | --- | --- | --- |
| CVA2- VP1 | TCAGTCCCATTCATGTCGCC | AATGCGTTGTTGGGGCATTG | 118 |
| Mouse β-actin | GTGCTATGTTGCTCTAGACTTCG | ATGCCACAGGATTCCATACC | 174 |
| Caspase9 | CTGCTGCGTGGTGGTCATTCTC | CACAATCTTCTCGACCGACACAGG | 109 |
| Caspase8 | GACTTTCTGCTGGGGATGGC | ATCGCTCTCTCAGGCTCTGG | 106 |
| Human β-actin | GGGCACGAAGGCTCATCATT | AGCGAGCATCCCCCAAAGTT | 285 |
| PE | TCCGGCCCCTGAATGCGGCTAATCC | ACACGGACACCCAAAGTAGTCGGTCC | 116 |
| EV71 | GCAGCCCAAAAGAACTTCAC | ATTTCAGCAGCTTGGAGTGC | 226 |
| CVA16 | ATTGGTGCTCCCACTACAGC | TCAGTGTTGGCAGCT GTAGG | 208 |
| CVA2**^*^** | CGGATGAAATTACCCAGCAAGCAAC | GCCCAATCGTTGTGAGTGGCAAG | 1003 |

Note: *; The full length of CVA2 VP1 genome.
