# Supplementary material for: Neonatal Murine Model of Coxsackievirus A2 Infection for the Evaluation of Antiviral Therapeutics and Vaccination
Source: Front Microbiol. 2021 May 28;12:658093. doi: 10.3389/fmicb.2021.658093 (PMC8192712; doi:10.3389/fmicb.2021.658093)
Supplement: Supplementary file 4 [file Table_2.DOCX]

**Table S2 Information of Clinical Specimens**

| Specimen# | Diagnosis | Gender | Age (month) | Admission time | PE | EV71 IgM | CRP |
| --- | --- | --- | --- | --- | --- | --- | --- |
| 1 | severe | female | 6 | 20170801 | + | - | + |
| 2 | severe | male | 8 | 20170628 | + |  | + |
| 3 | mild | female | 30 | 20170702 | + | - | + |
| 4 | severe | female | 34 | 20170804 |  | - | - |
| 5 | severe | female | 22 | 20170703 |  | - | - |
| 6 | severe | female | 34 | 20170702 |  | - | - |
| 7 | severe | male | 24 | 20170809 | + | - | - |
| 8 | mild | female | 6 | 20170705 | + | - | - |
| 9 | mild | male | 13 | 20170702 | + | - | - |
| 10 | mild | female | 36 | 20170801 | + | - | - |
| 11 | severe | male | 12 | 20170806 | + |  | - |
| 12 | severe | female | 62 | 20170801 | - | + | - |
| 13 | severe | male | 25 | 20170702 |  | + | - |
| 14 | mild | male | 28 | 20170607 |  | + | - |
| 15 | mild | male | 18 | 20170630 |  | - | + |
| 16 | severe | male | 48 | 20170617 |  | - | + |
| 17 | mild | male | 13 | 20170716 | - | - | - |
| 18 | mild | female | 55 | 20170623 |  | - | - |
| 19 | severe | male | 23 | 20170802 |  | - | - |
