# Supplementary material for: Neonatal Murine Model of Coxsackievirus A2 Infection for the Evaluation of Antiviral Therapeutics and Vaccination
Source: Front Microbiol. 2021 May 28;12:658093. doi: 10.3389/fmicb.2021.658093 (PMC8192712; doi:10.3389/fmicb.2021.658093)
Supplement: Supplementary file 5 [file Table_3.DOCX]

**Table S3 Analysis of RT-PCR Sequencing Results**

| Sample | Gene | Product | BLAST | Sequence ID | Homology (%) |
| --- | --- | --- | --- | --- | --- |
| 4 | CVA16 | 210 | Coxsackievirus A16 isolate  CVA16/SZ29/CHN/2014,  complete genome | KM215267.1 | 96(201/209) |
| 7 | EV71 | 231 | Coxsackievirus A2 VP1 gene for  polyprotein, partial cds, strain:  20134217-Sapporo-2013Dec | LC124143.1 | 96(216/224) |
| 8 | CVA16 | 212 | Coxsackievirus A16 isolate  CVA16/SZ29/CHN/2014,  complete genome | KM215267.1 | 96(202/210) |
| 11 | EV71 | 231 | Coxsackievirus A2 VP1 gene for  polyprotein, partial cds, strain:  20134217-Sapporo-2013Dec | LC124143.1 | 97(218/224) |
| 12 | EV71 | 231 | Coxsackievirus A2 strain 430895,  complete genome | JX867330.1 | 97(218/224) |
| 13 | EV71 | 228 | Enterovirus A71 strain  C4-NHRIH2011053-V12060359  1-TW-Nov-29-11 polyprotein  gene, partial cds | KF306099.1 | 98(222/227) |
| 14 | EV71 | 228 | Enterovirus A71 strain  C4-NHRIH2011053-V12060359  1-TW-Nov-29-11 polyprotein  gene, partial cds | KF306099.1 | 97(221/227) |
| 15 | CVA16 | 210 | Enterovirus A strain  S0098b/CA16/2013/CHN,  complete genome | KM402020.1 | 96(201/209) |
| 16 | CVA16 | 210 | Enterovirus A strain  S0098b/CA16/2013/CHN,  complete genome | KM402020.1 | 97(203/209) |
| 17 | CVA16 | 210 | Enterovirus A strain  S0098b/CA16/2013/CHN,  complete genome | KM402020.1 | 96(201/209) |
| 18 | CVA16 | 210 | Coxsackievirus A16 isolate  CVA16/SZ29/CHN/2014,  complete genome | KM215267.1 | 97(202/209) |
| 19 | PE | 115 | Coxsackievirus A16 isolate  CVA16/SZ29/CHN/2014,  complete genome | KM215267.1 | 99(113/114) |
| 7 | CVA2 | 1003 | Coxsackievirus A2 isolate CVA2/Shenzhen50/CHN/2012complete genome | KX595282.1 | 96  (918/955) |
| 11 | CVA2 | 1003 | Coxsackievirus A2 isolate CVA2/Shenzhen133/CHN  /2013,complete genome | KX595282.1 | 96  (923/960) |
| 12 | CVA2 | 1003 | Coxsackievirus A2 isolate CVA2/Shenzhen50/CHN  /2012,complete genome | KX595282.1 | 96  (915/954) |
